# Healing the Broken Heart; The Immunomodulatory Effects of Stem Cell Therapy

**DOI:** 10.3389/fimmu.2020.00639

**Published:** 2020-04-09

**Authors:** Marcus J. Wagner, Mohsin Khan, Sadia Mohsin

**Affiliations:** ^1^Independence Blue Cross Cardiovascular Research Center, Lewis Katz School of Medicine at Temple University, Philadelphia, PA, United States; ^2^Center for Metabolic Disease, Lewis Katz School of Medicine at Temple University, Philadelphia, PA, United States; ^3^Department of Physiology, Lewis Katz School of Medicine at Temple University, Philadelphia, PA, United States; ^4^Department of Pharmacology, Lewis Katz School of Medicine at Temple University, Philadelphia, PA, United States

**Keywords:** myocardial infarction, stem cell therapy, wound healing, immune response, immunomodulation

## Abstract

Cardiovascular Disease (CVD) is a leading cause of mortality within the United States. Current treatments being administered to patients who suffered a myocardial infarction (MI) have increased patient survival, but do not facilitate the replacement of damaged myocardium. Recent studies demonstrate that stem cell-based therapies promote myocardial repair; however, the poor engraftment of the transferred stem cell populations within the infarcted myocardium is a major limitation, regardless of the cell type. One explanation for poor cell retention is attributed to the harsh inflammatory response mounted following MI. The inflammatory response coupled to cardiac repair processes is divided into two distinct phases. The first phase is initiated during ischemic injury when necrosed myocardium releases Danger Associated Molecular Patterns (DAMPs) and chemokines/cytokines to induce the activation and recruitment of neutrophils and pro-inflammatory M1 macrophages (MΦs); in turn, facilitating necrotic tissue clearance. During the second phase, a shift from the M1 inflammatory functional phenotype to the M2 anti-inflammatory and pro-reparative functional phenotype, permits the resolution of inflammation and the establishment of tissue repair. T-regulatory cells (Tregs) are also influential in mediating the establishment of the pro-reparative phase by directly regulating M1 to M2 MΦ differentiation. Current studies suggest CD4+ T-lymphocyte populations become activated when presented with autoantigens released from the injured myocardium. The identity of the cardiac autoantigens or paracrine signaling molecules released from the ischemic tissue that directly mediate the phenotypic plasticity of T-lymphocyte populations in the post-MI heart are just beginning to be elucidated. Stem cells are enriched centers that contain a diverse paracrine secretome that can directly regulate responses within neighboring cell populations. Previous studies identify that stem cell mediated paracrine signaling can influence the phenotype and function of immune cell populations *in vitro*, but how stem cells directly mediate the inflammatory microenvironment of the ischemic heart is poorly characterized and is a topic of extensive investigation. In this review, we summarize the complex literature that details the inflammatory microenvironment of the ischemic heart and provide novel insights regarding how paracrine mediated signaling produced by stem cell-based therapies can regulate immune cell subsets to facilitate pro-reparative myocardial wound healing.

## Introduction

Cardiovascular disease (CVD) is a leading cause of mortality and health care expenditures within the United States (1). The total health care expenditure to treat patients with CVD within the United States alone is expected to exceed 1 trillion dollars by the year 2035 (2). Currently, one of the leading contributors of CVD are ramifications from sudden cardiac events, i.e., acute myocardial infarction (AMI). Current therapies being administered within the clinic, such as beta-blockers, diuretics, vasodilators, angiotensin converting enzyme (ACE) inhibitors, left ventricular assisted devices (LVADs), pacemakers, defibrillators, and/or stents (3, 4), have significantly increased patient survival outcome and can slow heart failure progression (3, 5). Unfortunately, these therapies do not prevent or reverse the activation of a deleterious cycle that perpetuates adverse cardiac remodeling resulting in depressed cardiac function and heart failure progression (3, 6–9). Consequently, there are an increasing number of patients who are now faced with long-term health burdens associated with AMI and heart failure pathology. Therefore, the ability to identify novel therapies that can jump start regenerative processes to replace damaged myocardium following a myocardial infarction (MI) is of tremendous urgency.

Recently, cell-based therapies targeting the differentiation of resident cardiac stem cell populations or the transplantation of multiple allogenic stem cell types to give rise to new cardiomyocytes that can replace the damaged myocardium following an MI has been extensively studied (7, 10–17). However, the efficacy of cell delivery, engraftment, and differentiation of these populations within the infarcted myocardium has proven to be challenging and a major limitation of cell-based therapy (3, 18–22). Despite these limitations, cell therapy has demonstrated the efficacy to elicit improved cardiac function and limit the adverse cardiac remodeling processes that occur following an AMI event (7, 23, 24). Current theory suggests stem cell populations are enriched epicenters that secrete a vast majority of factors such as: cytokines, chemokines, exosomes, and miRNAs enriched within exosomes. These signaling factors retain the ability to directly mediate the injured microenvironment of the infarcted heart without direct cell-to-cell contact; this theory is commonly referred to as the paracrine hypothesis (25). The direct effects of stem cell secretome on other cell types within the infarcted heart is under intense investigation (3, 7, 26, 27).

A MI results in the formation of a sterile wound that encompasses the ischemic tissue. During ischemic injury, a massive inflammatory response is mounted within hours of injury onset (28). This diverse inflammatory response proves essential in mediating the homeostasis of the cardiac tissue following injury onset by aiding in the clearance of necrotic tissue and the establishment of the reparative phase that prevents cardiac rupture. Although the inflammatory response is essential for cardiac repair, the chronic activation of such processes can contribute to the activation of a deleterious inflammatory cycle that can contribute to further heart failure pathology (21, 29), suggesting the suppression of the inflammatory microenvironment of the post-MI heart could be therapeutic. However, preliminary studies highlight that the broad immunosuppression of the immune system post-MI does not provide therapeutic potential (30), but rather the specific targeting of different immune cell subsets are more efficacious at promoting cardiac wound healing post-MI (30–34).

Due to the complexity and interplay of the innate and adaptive immune responses following tissue injury, finding a therapy that can properly orchestrate the direct targeting of different immune cell subsets has proven to be of great challenge. Most therapies to date have targeted the structural and functional adverse cardiac remodeling processes rather than the immunological response that regulates sterile wound healing. This allows for the development of a new and exciting field of research that can incorporate novel immune based therapeutics to mediate cardiac wound healing following AMI. Given transplanted stem cell populations have to endure the harsh inflammatory microenvironment of the infarcted heart (35–39), understanding the interplay between the adoptively transferred stem cell populations and the immune response could identify novel mechanistic and therapeutic targets that can regulate cardiac wound healing following ischemic injury. In this review, we will outline the individual role of immune cell subset populations in mediating cardiac repair following sterile, ischemic injury. We will further provide novel mechanistic insights highlighting how cell-based therapies can be used as a therapeutic target to regulate the inflammatory microenvironment of the infarcted heart, ultimately leading to improved myocardial wound healing.

## Immune System Response to Myocardial Infarction

During a MI, a coronary artery becomes occluded which compromises blood flow to the myocardium downstream of the occlusion site resulting in the deprivation of oxygen and nutrients, ultimately contributing to cardiomyocyte death via necrosis. Myocardium necrosis causes the activation of a triphasic inflammatory response. During the first phase, the alarmin phase (30), Damage Associated Molecular Patterns (DAMPs), such as heat shock proteins, extracellular RNA, TNFα, IL-1α, IL-1β, CXCL8, IL-18, IL-6, CCL2, and CCL7 are released from the injured myocardium (40–48). In approximately 12–24 h post-infarction, the DAMPs released from the necrosing myocardium reach and initiate the migration and extravasation of leukocyte populations (neutrophils, monocytes, and macrophages) into the infarcted tissue, marking the second phase of the post-MI inflammatory response. The immune cell response and specifically the cytokine inflammatory response evoked within the recruited immune cell subset populations is highly dependent on DAMP binding to Toll Like Receptors (TLRs) (49, 50). During the second wave, additional pro-inflammatory signals are secreted from the recruited leukocyte populations as these populations, specifically neutrophils and MΦs, facilitate the phagocytosis and clearance of the necrosed cardiomyocytes (30, 51, 52). During the third phase, anti-inflammatory M2 MΦs (52–57) and Tregs infiltrate into the infarcted myocardium and promote the removal of the initial pro-inflammatory leukocyte infiltrate to establish the pro-reparative phase that permits scar formation, myofibroblast proliferation, and neovascularization of the injured myocardium (53, 58). Each innate immune cell subset recruited during the second phase of the inflammatory response facilitates a distinct and essential role in preparing the injured tissue for the pro-reparative phase. If the temporal regulation of the pro-inflammatory and anti-inflammatory, pro-reparative phases are shortened or prolonged, cardiac wound healing is greatly affected (59, 60). Previous reports have identified that TLR signaling, specifically through TLR2 and TLR4 bolster further tissue injury following ischemic injury by perpetuating continued pro-inflammatory signaling and preventing the establishment of the pro-reparative phase (61, 62). The distinct role of immune cell subsets that have been shown to regulate cardiac wound healing are more clearly defined in the subsequent sections of this review.

### Innate Immune Response

As outlined above, a massive immune response that incorporates multiple immune cell subsets from both the innate and adaptive immune systems are activated and migrate into the infarcted myocardium to orchestrate the clearance of necrotic tissue and facilitate tissue repair. Some of the first immune cells to arrive within the tissue are encompassed within the innate immune response. The modulatory role of innate immune cell subsets in cardiac wound healing are well-characterized and outlined below. Although each immune cell subset is reviewed as a homologous, single step process, it is essential to remember the immune response during cardiac repair is an extensive, heterogenic response.

#### Neutrophils

Within hours of ischemic injury onset, the DAMPs, cytokines, and chemokines released from the damaged myocardium trigger the activation and recruitment of neutrophils, making them the first immune cell subset to respond to cardiac injury (1, 7, 58, 63–66). Neutrophils respond to DAMPs and CXC chemokine gradients that contain the glutamic acid-leucine-arginine motif, which encompasses the following factors: TNFα, interleukin 1β (IL-1 β), IL-6, and IL-18 (67–69). Upon arrival to the infarcted tissue, neutrophils will release granules (70) or undergo a process referred to as respiratory burst (1, 71). During these processes, degradative proteins, i.e., myeloperoxidase (MPO), lactoferrin, matrix metalloproteinase 9 (MMP9), and reactive oxygen species, are released into the infarcted myocardium and begin to facilitate the degradation and removal of necrotic tissue (72). Although the extracellular molecules released during degranulation and respiratory burst can directly interact with the viable myocardium and can cause additional myocardial damage, these pro-inflammatory processes are necessary for proper and efficient removal of the necrosed myocardium. This allows for the preparation of the tissue for the pro-reparative phase, which permits extracellular matrix maturation and scar formation to prevent cardiac rupture (1, 73–75).

In addition to releasing degradative enzymes and causing additional pro-inflammatory stimuli, neutrophils have also been reported to remove necrotic tissue through mechanisms of phagocytosis (73, 74). Neutrophils that are recruited toward the end of the pro-inflammatory phase have been reported to play an essential role in tempering the initial pro-inflammatory immune response (28). These late stage neutrophils will release signals such as lipoxins, resolvins, annexin A1, and lactoferrin to stop the migration of neutrophils to the infarction site and promote neutrophil clearance via apoptosis (76–78). During apoptotic processes, neutrophils increase their expression of decoy/scavenger receptors to deplete the infarcted tissue of pro-inflammatory chemoattractants (76, 78). Lastly “eat-me signals” will be expressed on apoptotic neutrophils to signal their clearance from the infarcted tissue via MΦ mediated phagocytosis (76, 78).

#### Macrophages and Monocytes

Macrophages are a heterogenous population that either take residence within the myocardium during embryonic development or influx into the myocardium following ischemic injury (7, 79). Cardiac tissue resident MΦs are believed to either arise from non-hematopoietic precursor cells within the yolk sac or from hematopoietic stem cells occupying the fetal liver/bone marrow (79). Currently, the exact origin of these populations are under extensive investigation. Chemokine receptor 2 (CCR2) negative populations are considered resident MΦs and do not rely on a signaling axis to mediate their recruitment into the injury site, whereas MΦs derived from circulating monocytes positively express the CCR2 receptor and rely on the monocyte chemoattractant protein-1 (MCP-1)/CCR2 signaling axis to regulate their recruitment into the infarcted tissue immediately following injury onset (46).

The macrophage response to myocardial infarction is classified into two categories: 1) pro-inflammatory, classically activated M1 MΦs, or 2) anti-inflammatory, alternatively activated M2 MΦs. Pro-inflammatory, classically activated M1 MΦs contain high expression of lymphocyte antigen 6 complex (Ly6c) and is the most prominent MΦ population occupying the infarcted tissue about 3–4 days post ischemic injury onset (47, 53–56). These M1 populations contain proteolytic enzymes (cathepsins and MMPs) and a pro-inflammatory secretome enriched in IL-1β, TNFα, nitric oxide (NO), and IL-6 (32, 47, 55, 80, 81). Ultimately, M1 MΦs work in parallel with neutrophils to maintain a harsh inflammatory state to induce the degradation and clearance of necrotic tissue. M1 MΦs help facilitate the removal of necrotic tissue and apoptotic neutrophils by phagocytic processes (1, 46, 56). The phagocytosis of neutrophils is believed to help induce the transition of the pro-inflammatory, phagocytic M1 MΦs into anti-inflammatory, pro-reparative M2 MΦs (1, 82). Other cellular compartments, specifically Tregs, are also believed to help facilitate the M1 to M2 MΦ conversion (1). In humans, the phenotype of monocytes and their pro- verse ant-inflammatory properties are defined based on the expression of two cell surface markers: CD14 and CD16 (83, 84). Approximately 80–90% of human monocytes retain CD14++(high) and CD16- expression, analogous to the Ly6C^hi^ populations observed within murine species. CD14++, CD16- monocytes retain the pro-inflammatory M1 MΦ signature (85–87). Infarct size and left ventricular function is negatively correlated with the amount of CD14++, CD16- monocytes present within the myocardium post-AMI (88–90). Unlike the M1 and M2 MΦ dichotomy described in murine models of cardiac injury, a third subset of monocytes are defined as having CD14++ (high) and CD16+ expression (86, 91). The direct inflammatory role of this monocyte population is of great discrepancy (87, 92). Some groups have identified these MΦ populations as retaining a pro-inflammatory signature marked by TNFα secretion when stimulated with LPS, while other groups believe this population is a pre-mature M2 MΦ cell type (87, 92).

M2 MΦs express low levels of Ly6C and are the most prominent MΦ population occupying the infarcted tissue at 5–7 days post ischemic injury onset (47, 93–95). The M2 MΦ population contains an anti-inflammatory secretome that is enriched in IL-10 (28, 55, 57, 58, 96), transforming growth factor β (TGF-β) (97), vascular-endothelial growth factor (VEGF) (55, 57, 98), insulin-like growth factor 1 (IGF-1), platelet-derived growth factor α (PDGFα), and fibronectin. This secretome helps establish the anti-inflammatory, pro-reparative stage which allows for the initiation of pro-angiogenic and pro-reparative processes (57, 99). Depletion of M2 MΦs greatly hinders cardiac repair, resulting in an increased incidence of cardiac rupture (1, 57). In humans, M2 monocytes possess CD16+ and CD14+ (low) expression and have been shown to facilitate pro-reparative processes similar to ther murine Ly6c^lo^ expressing counterparts (83).

Numerous reports have elucidated that MΦs can be directly coupled to cardiac regeneration. The ability of the neonatal heart to regenerate following cardiac injury is closely coupled with resident MΦ populations occupying the injured tissue. The ablation of MΦ populations within the neonatal heart hinders its ability to regenerate following cardiac injury (1). Ideally, identifying novel therapeutic targets that repress pro-inflammatory MΦ populations, but expand tissue resident MΦs derived from embryonic progenitors could help increase cardiac repair following ischemic injury (7).

#### Toll Like Receptors Mediate Immune Cell Response to Injury

Toll like receptors directly mediate the cytokine signaling mounted by immune cell subsets of both the innate and adaptive immune responses. Previous studies have shown DAMP signaling through TLR2 (61), TLR3 (100), TLR4 (61), and TLR9 (61) can further perpetuate cardiac injury by provoking an enhanced pro-inflammatory response. Identifying the distinct role of these individual signaling modalities and therapies that can directly modulate signaling through these receptors during cardiac injury is under extensive investigation and can have immense impact in mediating immune cell phenotype, response, and function in the context of ischemic cardiac injury (101).

### Adaptive Immune Response

The post-MI innate inflammatory response has been characterized extensively; but to date, little is known regarding the adaptive immune response (32, 102–104). Preliminary studies have identified a chronic activation of inflammatory pathways in heart failure patients, suggesting that chronic inflammation can be directly coupled with heart failure pathology (7, 32, 105). Understanding the mechanisms that regulate the adaptive immune response, specifically the T-lymphocyte response, can lead to the discovery of novel therapeutic targets that can improve the long-term clinical outcomes for patients post-MI.

#### T-Lymphocytes

T-lymphocyte populations are classified as either a cytotoxic (CD8^+^) or T-helper (CD4^+^) cell population (1, 106, 107). Regardless of cell type, CD8 and CD4 T cell populations have been shown to influx into the injured myocardium during the reparative phase of the cardiac repair cycle (32, 64, 97, 102, 108, 109). Yang et al. was the first group to identify that T cells contribute to reperfusion injury (I/R) post-MI (1, 110). Yang's group reported that mice deficient in producing viable T-cell populations (RAG1 KO mice) possess a smaller lesion size post-MI and the subsequent introduction of T-lymphocytes from wild type mouse donors results in increased injury severity (110). This seminal report indicates that T cell populations can greatly hinder cardiac repair. However, there is great heterogeneity within T-lymphocyte subset populations in regard to T cell function, antigen recognition, and response to cardiac injury. Identifying the individual roles of T cell populations in response to cardiac injury proves specific to the subset type and the environmental context in which the subset resides. For example, the T-regulatory cell (Treg), a specific CD4+ T cell subset, has shown to exhibit cardioprotective properties post-MI, as the depletion of this population greatly compromises cardiac repair (111, 112). Contrary to these therapeutic reports, other studies have reported Treg specific depletion can reduce infarct size (113). The controversial literature surrounding Treg phenotype and its function in the context of cardiac repair is explored more in the “T-regulatory Cells” section of this manuscript.

The mode of T cell activation and the paracrine microenvironment surrounding T cell populations resident within the injured cardiac muscle or draining lymph node can greatly modulate the activation state and phenotype of T cell populations. T cells can only recognized antigen peptide when presented in a major histocompatibility complex (MHC), which are commonly expressed on professional antigen presenting cells (i.e., dendritic cells and macrophages). CD4 T cells can only recognize peptides presented in a MHCII complexes, while CD8 T cells can only recognize peptides presented in a MHCI complex (106, 107, 114). The presentation of these antigens through these complexes will cause T cells to become activated and mount an immune response against the presented antigen/peptide. During cardiac injury, such as an MI, tissue proteins from the myocardium are released into the systemic circulation which can then enter the draining lymph nodes. These proteins are then processed by antigen presenting cells and presented to T cell populations residing within the lymph nodes via MHC complexes. Since these antigens are not present in the thymus during development, tolerance to the antigen cannot be achieved and will result in the activation of T cell populations, which causes a chronic autoimmune response against the myocardium (1). Current reports have identified several cardiac autoantigens that are released from cardiomyocytes during ischemic injury. Several of these antigens have been linked to proteins associated with the cardiomyocyte, specifically cardiac troponin I and myosin heavy chain (102, 115). Oral administration of these antigens to induce immunological tolerance have proven to help reduce myocardial injury in various cardiovascular disease models, including myocardial infarction (116–120).

The priming of T cell populations with different paracrine stimuli has also been shown to regulate the phenotypic signature and migratory response of T cell populations. For example, priming T-cell populations with hepatocyte growth factor (HGF) can induce cardiotropism, by upregulating homing receptors that can induce T cell migration to the cardiac muscle. Identifying therapeutic interventions that can mediate T cell phenotype by regulating antigen presentation or paracrine signaling could substantially impact the adaptive immune response to cardiac injury and repair (1, 121).

#### B-Lymphocytes

Another lymphocyte population that is encompassed within the adaptive immune system and has been poorly investigated in mediating cardiac repair processes are B-lymphocytes. Upon myocardial injury, B cells have been reported to influx into the myocardium during the reparative phase, but the exact mechanisms/factors that mediate their activation and response to cardiac injury are poorly characterized and not well-understood (64). Preliminary studies that have depleted B cell populations during ischemic injury have reported marked improvements in myocardial recovery post-MI (48). Seminal reports have proposed that the infiltrating B cells into the injured myocardium release autoantibodies that cause further injury to the ischemic myocardium (122, 123). Specific autoantibodies that are produced against the cardiac muscle following ischemic injury include antibodies that are reactive to contractile proteins retained within cardiomyocytes during the homeostatic state, but are released into the periphery upon myocardial injury (i.e., actin, myosin heavy chain, and cardiac troponin I) (122, 124, 125). Autoantibody generation has been reported in the following cardiac diseases: ischemia IR/MI (126, 127), chronic heart failure (123) Chagas disease (128), and autoimmune myocarditis (129). Identifying the mechanisms that mediate B cell activation and their response to myocardial injury can lead to the identification of new immunomodulatory therapies that can promote cardiac repair processes.

### T-Regulatory Cells

T-regulatory cells, commonly referred to as Tregs, are notorious for their immunosuppressive properties and in establishing T cell tolerance to foreign antigen presentation outside of the initial developmental and selection processes that occur within the thymus (130–133). Tregs are best characterized by their expression of the Forkhead box P3 (FoxP3) transcription factor. Current literature surrounding the inflammatory role and other physiological processes of Tregs during cardiac injury is inconsistent and warrants further investigation. Initial studies identified that Tregs facilitate a cardioprotective role during ischemic injury, as Treg cell depletion during MI greatly hinders cardiac repair processes (24, 33, 111, 134–136) and contributes to a reduced survival rate, increased adverse cardiac remodeling/function, and larger infarct sizes (32, 135). Subsequent studies that targeted the expansion of Tregs via CD28 superagonist antibody or through the adoptive transfer of Treg populations following a myocardial infarction have demonstrated the ability to increase cardiac repair and function (134–138).

In recent studies, Tregs have also been shown to mediate processes that are extrinsic to their immunosuppressive role in the peripheral immune system. Several groups have identified tissue specific resident Tregs that directly mediate the homeostatic processes of the specific tissues they populate (139), which is extensively reviewed in Panduro et al. Skeletal muscle Tregs contain a distinct T-cell receptor repertoire (TCR) that is only expressed on Tregs that influx into the injured skeletal muscle, not on systemic, peripheral Tregs occupying the systemic lymph nodes or spleen (140). Further investigation into the therapeutic role of the muscle Treg in mediating skeletal muscle repair has identified that the muscle Treg is necessary for facilitating the polarization of the Ly6c^Hi^, M1 MΦ populations to Ly6C^lo^, M2 expressing MΦ populations during skeletal muscle wound healing. The depletion of the muscle Tregs during skeletal muscle injury prevents the establishment of a pro-reparative phase and hinders skeletal muscle repair (140). In addition, skeletal muscle Treg mediated production of amphiregulin has been shown to directly induce the expansion and differentiation of satellite progenitor cells to form new myotube structures and increase skeletal muscle regeneration/ healing processes (140). Whether a distinct tissue Treg population occupies the heart and specifically mediates myocardial repair following ischemic injury has not yet been classified.

Contrary, to the reports outlined above, other groups have reported that Treg cells can also contribute to pathological remodeling of the tissues they populate. One specific study demonstrates Treg specific depletion can reduced infarct size in an I/R injury model (112). These claims can be further confirmed by Bansal et al. which have identified that Tregs have phenotypic plasticity and can revert to a pathological, TNFα+ producing phenotype during chronic ischemic injury (141). TNFα, Foxp3^+^ Tregs at 8 weeks post permanent ligation of the left anterior descending artery (LAD) contribute to compromised immunosuppressive properties, an increase in infarct size, and decreases in cardiac function. However, temporary depletion of these Tregs and the adoptive transfer of Tregs from non-infarcted animals can revert this pathogenic phenotype and provide therapeutic effects allowing for increased cardiac repair (141). These seminal reports identify the presence of molecular triggers that can cause Treg cell plasticity and can greatly affect cardiac repair processes. Identifying these molecular triggers and therapies that support an anti-inflammatory, pro-reparative phenotype can yield therapeutic targets that can greatly enhance cardiac repair.

## Cell-Based Therapies Mediate Cardiac Repair via Immunomodulatory Processes

Given the cardiac repair process is tightly coupled to the inflammatory microenvironment of the injured tissue, the ability to mediate the transition between the pro-inflammatory state, and the anti-inflammatory, pro-reparative state has been the center of prevailing investigation (105). Numerous clinical studies have incorporated the usage of broad immunosuppressive agents to reduce the inflammatory response following myocardial infarction and other cardiac diseases in hopes to reduce myocardial injury and increase cardiac repair. Unfortunately, results from these clinical approaches using a variety of different drugs, have led to the conclusion that the broad suppression of the inflammatory response during cardiac repair does not improve cardiac wound healing (18, 19, 142–145). A review of the immunosuppressive agents tested within the clinic and their clinical outcomes are further reviewed in Huang et al. These studies emphasize that the broad immunosuppression of an interconnected, multilayered immune response is not the appropriate approach to mediate the cardiac repair process, but rather distinct regulatory mechanisms that can target specific immune cell populations is more efficacious in promoting cardiac repair.

**Figure 1:**
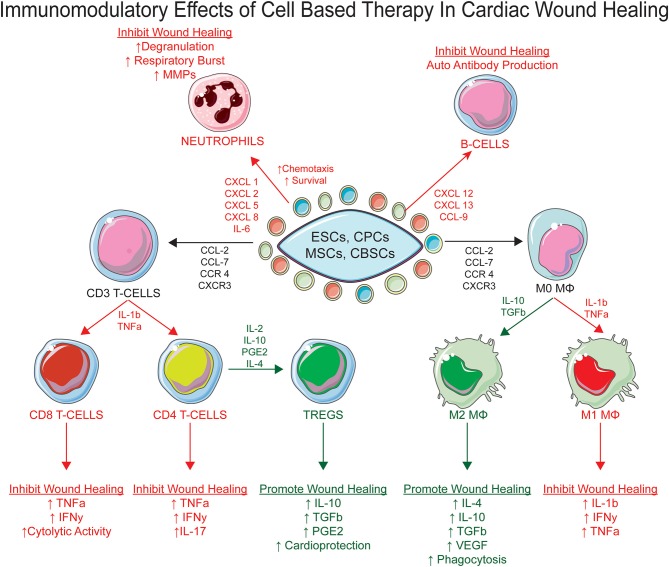
The immunomodulatory effects of stem cell therapy post myocardial infarction. The paracrine secretome produced by cell based therapy can directly mediate the inflammatory microenvironment of the infarcted heart by acting on multiple immune cell subsets to promote myocardial wound healing, specifically neutrophils, MΦs, T-Cells, and B-Cells.

Cell-based therapies were originally tested in large animal models and clinical studies with the hope that the allogenic transfer of stem cell populations into the injured myocardium would give rise to new cardiomyocyte formation to replace the cardiac tissue lost during ischemic injury (7, 146). The most common cell therapies that have been tested within the clinic incorporate the introduction of autologous stem cell populations derived from the bone marrow or cardiac tissue and reintroduced into the infarcted heart, a process commonly referred to as the adoptive transfer of autologous stem cell populations (7, 14, 147). Most recent clinical evidence have indicated that the adoptive transfer of stem cell populations into the ischemic heart provides modest functional improvement (7). Original hypothesizes purposed that the adoptively transferred stem cell populations would engraft within the injured myocardium and differentiate into new cardiomyocytes (11, 12, 14, 147). However, recent reports have identified that the transdifferentiation of stem cells into new cardiomyocytes from the adoptively transferred stem cell populations is a rare occurrence and not the main reparative mechanism of cell based therapies within the infarcted myocardium regardless of the cell type used (7, 27, 148). Stem cells have shown to produce many different types and quantities of “growth factors, cytokines, microRNAs, and exosomes to modify their surrounding microenvironment”, a theory commonly referred to as the paracrine hypothesis (25, 149–153). Given that the injected stem cell populations lies in close proximity to recruited immune cells within the injured myocardium, it is reasonable to propose that stem cell populations can directly signal to and modify the recruitment, activation, phenotype, and function of distinct immune cell subsets (35–39). Factors released from stem cells have been shown to directly interact with neutrophils, MΦs, T-cells, and B-cells (Figure 1) (149, 150, 154–156). Given stem cells can directly orchestrate different immune cell processes without providing broad immunosuppression of all immune cell populations, cell-based therapy could be the ideal therapeutic that provides the appropriate level of immunosuppression of pro-inflammatory inducing immune cells, while expanding immune cell populations that contribute to the establishment and maintenance of the pro-reparative state.

Several cell types have been introduced to the injured myocardium and have reported varying levels of myocardial repair; therefore, it is reasonable to propose the immunomodulatory effects of cell-based therapy is highly dependent on cell type (3, 7, 19, 157–161). In the subsequent sections of this review we will summarize the status of cell therapy in modulating the immune response after myocardial injury.

### Immunomodulatory Properties of Stem Cells

Numerous stem cell types have been introduced into the infarcted heart to help reduce ischemic injury, however which cell type has superior therapeutic effects regarding myocardial wound healing is not clearly identified. Since the discovery that Mesenchymal Stem Cells (MSCs) contain immunosuppressive properties, the mechanisms that mediate their immunomodulatory ability have been thoroughly studied and has helped identify essential mechanistic studies that must be tested within other cell lines to asses their immunomodulatory capacity, specifically Cardiac Derived Cells (CDC's) (162–165), Embryonic Stem Cells (ESCs) (163), and Cortical Bone Derived Stem Cells (CBSCs) (166–168). In general, stem cells have demonstrated the ability to mediate the inflammatory microenvironment of the infarcted heart via an enriched paracrine secretome and cell membrane receptors that can directly and indirectly mediate the chemotaxis, apoptosis, immunosuppression, and phenotype polarization of immune cell subsets elicited during cardiac repair. In sections “Stem Cells and Neutrophils” to “Stem Cells and B Cells,” we will identify how stem cell mediated paracrine signaling can directly modulate immune cell response following ischemic cardiac injury.

#### Stem Cells and Neutrophils

One of the first immune cells recruited to the injured myocardium is the neutrophil (58, 63–66, 169, 170). Several reports have demonstrated that MSC secretome is enriched in chemotactic signaling molecules, specifically CXC Chemokine Ligand 1 (CXCL1), CXCL2, CXCL5, and CXCL8, which directly mediate the recruitment and retention of neutrophils within the infarcted heart (171–177). In addition, when neutrophils are co-cultured with MSCs, neutrophil viability is increased (178–180). The therapeutic effect of MSCs in promoting neutrophil survival is believed to be provided by MSC mediated secretion of IL-6, for the neutralization of IL-6 in MSC/neutrophil co-cultures abolishes neutrophil viability (178). Given MSCs support neutrophil recruitment and survival, this raises a concern that MSCs may not be an optimal cell type to mediate cardiac repair, as this cell type supports a sustained pro-inflammatory state by increasing the recruitment and retention of neutrophils within the infarcted myocardium.

#### Stem Cells and MΦs

Several studies have demonstrated the effects of stem cells on macrophage populations after cardiac injury. The MSC secretome has demonstrated the ability to directly mediate the M1 to M2 MΦ polarization within the infarcted heart by inhibiting the pro-inflammatory effects of the M1 MΦs and supporting the formation of M2 MΦs (181). When MΦs are co-cultured with MSCs, the medium contains less pro-inflammatory induction signals expressed by M1 MΦs, specifically TNFα, IL-1β, IL-6, IFNγ, and IL-12; but increases the anti-inflammatory, pro-reparative cytokine expression: TGFβ and IL-10 (175, 182, 183). The MSC secretome also contains enriched expression of Prostaglandin E2 (PGE2), IL-1Rα, and TGF-β (175, 181, 184), which have been shown to facilitate M1 to M2 MΦ polarization. In addition to mediating MΦ polarization processes, MSCs are also believed to mediate MΦ recruitment to the injured myocardium via the secretion of the following MΦ chemotaxis signaling molecules: CCL-2, CCL-7, and CCL-12 (185–187).

Studies have also reported that cardiac derived cells (CDCs) can directly mediate cardiac wound healing processes via paracrine and exosome dependent signaling mechanisms (188–190). CDC derived exosomes have been shown to mediate M1 to M2 MΦ polarization (32, 54, 191, 192). Intracoronary injection of CDC derived exosomes results in an increase in M2 MΦ expression and decreased pro-inflammatory gene expression (193). Recent studies surrounding ESCs identify that these populations are not considered to be immune privileged as the adoptive transfer of primitive ESC populations elicits a massive inflammatory response and can consequently result in the rejection of the administered cell therapy (133, 194–201). ESCs have been shown to increase MHCI and MHCII expression within the heart, whether these cells were recruited to the heart or underwent *de novo* generation from ESCs has not been clearly defined (194). Given the primitive nature of ESCs and their superior differential abilities, most of the immunomodulatory work using ESCs is via the manipulation of central tolerance by ESC-derived hemopoietic stem cell establishment (202–205). Myeloid cells are a key therapeutic target given their ability to regulate the initial and prolonged inflammatory responses. Initial studies suggested ESCs can differentiate into either M1 or M2 MΦ populations and subsequently alter the inflammatory response (206). In a study by Kudo et al. an ESC derived suppressor cell line that contains an M1/M2 MΦ phenotype hybrid was generated and demonstrated the ability to mediate T cell response and permit cardiomyocyte engraftment in a nitric oxide (NO) dependent manner (194). Immune suppression is essential for ESC engraftment, however the heterogeneity that can occur from ESC derived immune cell populations could prove problematic and needs to be better optimized.

Direct intramyocardial injection of Cortical Bone Derived Stem Cells (CBSCs) into infarcted myocardium immediately following ischemia reperfusion results in the marked increase in (5-Ethynyl-2-deoxyuridine) Edu+ cells that predominantly express CD45 and von Willebrand factor, suggesting that CBSCs mediate wound healing processes by directly modulating the leukocyte inflammatory response to MI, rather than the regeneration of new cardiomyocytes (7, 167). CBSCs contain a paracrine secretome that is enriched in growth factors that have been reported to be cardioprotective (7, 207, 208). CBSCs express low levels of factors that elicit pro-inflammatory responses, which explains the increased prevalence of M2 MΦ expression in CBSC treated animals post-IR (168).

#### Stem Cells and T Cells

MSCs can directly regulate the activation and proliferative state of T Cell populations by direct cell to cell contact via the expression of co-inhibitory signaling molecules. Reports have identified that MSCs express co-inhibitory signaling ligands on their surface, specifically Fas ligand (FasL) and TNF-Related Apoptosis-Inducing Ligand (TRAIL). Once FasL and TRAIL expressed on the cell surface of MSCs encounters their complementary receptors on the surface of the T cell, apoptotic processes are induced (209, 210). This regulatory mechanism directly prevents T cell expansion within the infarcted myocardium and can directly downregulate the amount of pro-inflammatory T cell subset populations resident within the infarcted myocardium, which in turn promotes the establishment of the pro-reparative state. MSCs also contain an enriched secretome that can mediate the phenotype, proliferation, and activation state of T cell populations without requiring direct cell to cell contact. The MSC secretome is enriched in inducible NO synthase (iNOS), Indoleamine-Pyrrole 2,3-Dioxygenase (IDO), TGF-β, and PGE-2. All of these paracrine factors have demonstrated the ability to directly prevent T cell proliferation (171, 211–213); in turn this would explain why T cell populations arrest in G_0_ when co-cultured with MSCs (214, 215). As previously outlined above, halting the proliferative capacity of pro-inflammatory T-cell subsets limits the impact of a chronic pro-inflammatory microenvironment within the infarcted heart.

MSCs have also been shown to regulate the proliferation of T-conventional (Tconv) cell populations indirectly by enhancing the immunosuppressive capabilities of T-regulatory cell populations. As previously outlined in the “T-regulatory Cells” section of the present manuscript, the main immunosuppressive cell within the adaptive immune system is the Treg cell. The overall Treg signature, specifically the immunosuppressive properties of the Treg are considered to be plastic and can be significantly influenced by external paracrine signals. The exact signals that mediate the immunosuppressive and functional plasticity of Treg populations is elusive and under intense investigation. The co-culturing of MSCs with pan T-lymphocyte populations can induce the expansion of FoxP3^+^ Treg populations (197, 216, 217). This induction is believed to be mediated by TGF-β and PGE2 paracrine signals which are retained within the MSC secretome. Exposing Treg cells to the MSC secretome has also shown to enhance the immunosuppressive capabilities of Treg cells as marked by increased IL-10 production and the increased expression of the programmed cell death 1 (PD-1) receptor (218).

Human cardiac progenitor cells populations (hCPCs) have also been reported to repress Th1/2^+^ cell expression and promote Treg cell proliferation, expansion, and immunosuppressive function via PD-1 dependent expression (219). ESCs have also demonstrated the ability to increase the recruitment of CD3^+^ T cell populations into the injured myocardium and subsequently induce Treg cell formation, however there is great plasticity within the Treg cell populations when exposed to ESCs, resulting in a heterogenic response (220–222).

Further investigation into the increased CD45 compartment of CBSC treated hearts, previous described above, identify that CD4+ T cellular compartments are significantly increased within the hearts of animals treated with CBSCs post-IR (7, 168). As previously mentioned above, CBSC secretome is enriched in IL-2, IL-4, IL-10, and TGF-β, all of which are cytokines that can directly mediate Treg cell formation and function. Whether CBSCs' can directly induce Treg cell formation or regulate the immunosuppressive abilities and formation of such populations has not yet been reported.

#### Stem Cells and B Cells

The ability of the MSC secretome to mediate B cell populations and their function in the context of cardiac repair is poorly documented compared to the previously outlined immune cell subsets (154). Like T Cells, the co-culturing of MSCs with B Cell populations abolishes B cell proliferation with B cells arresting in the G_0_/G_1_phase (223). In addition, a seminal report concluded that the MSC secretome can halt B cell maturation, marked by decreased expression of CD138, IgG, IgA, and IgM (223, 224). To date, no studies have fully elucidated the direct effect ESCs have on B-cell maturation or function in the context of MI. However, ESCs were incorporated to directly modulate central tolerance to injury via the establishment of ESC-derived hematopoietic stem cells (202–205). Initial studies have identified ways to promote and mediate the differentiation of ESCs into M1 or M2 MΦ lineages (206). These ESC derived MΦ lineages can then interact with other immune cell subsets to mediate the post-MI inflammatory response (206). Whether ESCs can directly regulate humoral immunity within the infarcted heart by deriving specific B lymphocyte lineages or indirectly via ESC derived hematopoietic stem cells/macrophages is poorly understood and warrants further investigation (220, 221). The regulatory effect of ESC derived hematopoietic stem cells on lymphocyte populations, specifically on t-lymphocyte populations, has previously been outlined in the “Stem Cells and T Cells” section of the present manuscript (194).

## Conclusions and Future Directions

The cardiac repair process of the ischemic heart is closely coupled to a complex and interconnected inflammatory response that facilitates the clearance of necrotic tissue during early stages of cardiac repair and the establishment of an anti-inflammatory microenvironment that is supportive of pro-reparative processes. There is a delicate balance between these two inflammatory states, if either is prolonged or shortened cardiac wound healing can be greatly compromised. Understanding how individual immune cell populations can mediate cardiac repair will help identify novel therapeutics that can be used to orchestrate more effective cardiac wound healing following ischemic injury. Cell-based therapies were originally introduced to facilitate the replacement of damaged tissue via the transdifferentiation of autologous stem cells into viable myocardium. However, the transdifferentiation of autologous stem cells into new cardiomyocytes is considered a rare event, despite the therapeutic effects exhibited by cell-based therapies. Stem cells are enriched epicenters that contain a diverse paracrine secretome that can directly orchestrate cardiac repair processes amongst multiple cell types. Given the close proximity of engrafted stem cell populations to resident and recruited immune cell populations within the infarcted heart it is reasonable to propose that the paracrine factors secreted from these stem populations can directly mediate the activation, recruitment, function, and phenotype of immune cell populations that orchestrate cardiac repair. The therapeutic efficacy of cell-based therapy within the heart is contingent on the cell type that is used. Each stem cell type has varying attributes, such as isolation method, differentiation state, population heterogeneity, and overall efficacy in mediating cardiac repair. Therefore, the complete characterization of each cell types secretome on the inflammatory microenvironment of the infarcted heart is essential. Ideally, an optimum cell type will promote the differentiation and establishment of pro-reparative immune cell subsets, i.e., M2 MΦs, and Treg cells, and aide in the suppression of pro-inflammatory cells types, i.e., neutrophils, M1 MΦs, and Tconv cell populations, that can further contribute to additional injury. To date, studies have mainly focused on delineating the MSC secretome. Given the failed clinical efficacy of MSCs, it is essential to investigate the secretome of other stem cell populations. Investigating novel stem cell populations that are more primitive and exhibit an enhanced paracrine secretome that can further induce the transition of the inflammatory microenvironment of the infarcted heart from a pro-inflammatory to a pro-reparative state is necessary and would have immense therapeutic impact (7). Additional studies investigating whether stem cell populations can directly mediate autoantigen presentation of cardiac antigens released during ischemic injury could be of great therapeutic impact. Delineating how cell-based therapies can be optimized to mediate the inflammatory microenvironment of the infarcted heart is an essential need and requires collaborative investigation from cardiac physiologists, stem cell biologists, and immunologists.

## Author Contributions

MW wrote and prepared the current manuscript. MK and SM reviewed and aided in the preparation of the current manuscript. All authors read and approved the final manuscript prior to submission.

### Conflict of Interest

The authors declare that the research was conducted in the absence of any commercial or financial relationships that could be construed as a potential conflict of interest.

## Abbreviations

CVD: Cardiovascular disease
AMI: acute myocardial infarction
DAMPs: Danger Associated Molecular Patterns
MΦs: macrophages
Tregs: T-regulatory cells
ACE: angiotensin converting enzyme
LVADs: left ventricular assist devices
TNFα: tumor necrosis factor α
IL: interleukin
MPO: myeloperoxidase
MMP9: matrix metalloproteinase 9
CCR: Chemokine receptor
MCP-1: monocyte chemoattractant protein-1
NO: nitric oxide
Ly6c: lymphocyte antigen 6 complex
TGF-β: transforming growth factor β
VEGF: vascular-endothelial growth factor
IGF-1: insulin-like growth factor 1
PDGFα: platelet-derived growth factor α
I/R: ischemia/reperfusion
RAG1 KO: recombination activating 1 knock out
MHC: major histocompatibility complexes
HGF: hepatocyte growth factor
FoxP3: Forkhead box P3
TCR: T-cell receptor repertoire
LAD: left anterior descending artery
MSCs: mesenchymal stem cells
CDCs/CPCs: Cardiosphere Derived Cells
ESCs: Embryonic Stem Cells
CBSCs: Cortical Bone Derived Stem Cells
CDCs: cardiac derived progenitor cells
CXCL: CXC chemokine ligand
PGE2: prostaglandin E2
Edu+: 5-Ethynyl-2-deoxyuridine
FGF: fibroblast growth factor
Ang-1: Angiopoietin-1
FasL: Fas ligand
TRAIL: TNF-related apoptosis-inducing ligand
iNOS: inducible NO synthase
IDO: indoleamine-pyrrole 2, 3-dioxygenase
PD-1: programmed cell death 1 receptor
TLRs: Toll-Like Receptors
hCPCs: Human cardiac progenitor cells populations.
